# The prevalence and risk indicators of tooth wear in 12- and 15-year-old adolescents in Central China

**DOI:** 10.1186/s12903-015-0104-9

**Published:** 2015-10-09

**Authors:** Jing Zhang, Yangge Du, Zhao Wei, Baojun Tai, Han Jiang, Minquan Du

**Affiliations:** The State Key Laboratory Breeding Base of Basic Science of Stomatology (Hubei-MOST) & Key Laboratory of Oral Biomedicine Ministry of Education, School & Hospital of Stomatology, Wuhan University, Luoyu Road 237, Wuhan City, 430079 China

**Keywords:** Tooth wear, Adolescent, Risk indicators, Tooth attrition, Tooth erosion, Tooth abrasion

## Abstract

**Background:**

Tooth wear has been investigated in numerous countries, and the prevalence has varied. However, the data on tooth wear in China are scarce. The aim of this study was to describe the prevalence of tooth wear and to investigate the relative indicators associated with tooth wear in 12- and 15-year-old adolescents in Wuhan City, Hubei Province, Central China.

**Methods:**

A cross-sectional descriptive study was undertaken among 720 adolescents in Hubei Province, Central China. The age groups in this study were 12- and 15-year-old, and each group consisted of 360 participants in which females and males represented 50 % each. A modified version of the Basic Erosive Wear Examination (BEWE) tooth wear index was used for the buccal, cervical, occlusal/incisal and lingual surfaces of all of the teeth in the 720 adolescents. All of the participants were asked to answer a questionnaire consisting of questions about their current and historical dietary habits and oral hygiene.

**Results:**

The prevalence of tooth wear was 18.6 and 89.4 % in 12- and 15-year-old adolescents, respectively. The prevalence rates of dentin exposure were 1.9 and 5.6 %, respectively. A significantly higher prevalence of tooth wear and dentin exposure in 15-year-old adolescents was found than in 12-year-old adolescents (*p* < 0.001 and *p* = 0.011). Several factors such as drinking soft drinks and fruit juices immediately after sports, taking aspirin, reflux, unilateral chewing, tooth brushing once daily or less often, duration of brushing less than 2 min and swimming in the summer were found to be associated with tooth wear.

**Conclusions:**

Tooth wear in 12- and 15-year-old adolescents in Central China is a significant problem and should receive greater attention. The prevalence of tooth wear increases with age and associated with socio-behavioral risk factors.

## Background

Wear can be defined as the progressive loss of material from the contacting surfaces of a body, caused by relative motion on the surface [1]. Tooth wear has been used to describe the loss of hard tissue caused by mechanical and/or chemical processes without bacterial action [2]. Tooth wear is a complex, multifactorial phenomenon involving the interplay of biological, mechanical, chemical and tribological factors. It consists of three main mechanisms: abrasion, attrition and erosion. Abrasion is the pathological wear of dental hard tissue by abnormal mechanical processes. Attrition is the physiological wear of dental hard tissues due to friction between opposing dentition or restorations, and it is the most common type of wear [3] and is inevitable as a part of the normal aging process [4]. Erosion is tooth surface loss caused by chemical or electrochemical action. Furthermore, abfraction is another type of tooth wear, which consists of cervical lesions caused by fatigue wear. These etiological factors act synchronically or diachronically in the tooth wear process.

The prevalence of tooth wear varies widely in the general population. Numerous studies have revealed correlations between tooth wear and age, proving that the severity of tooth wear increases with age [5–7]. In addition, with the aging tendency of the population, the incidence of natural tooth retention has increased, causing a greater prevalence of tooth wear in aging people. In northwest China, the prevalence of tooth wear in aging people ranged from 85.51 to 100.0 %, based on the site of teeth [8]. Nevertheless, this finding does not mean that the prevalence of tooth wear in adolescents can be ignored. In Birmingham, UK, 48 % of 14-year-old children had low erosion, 51 % had moderate erosion, and 1 % had severe erosion [9]. In Brazil, among 295 12-year-old adolescents, the prevalence of dental wear was 26.90 % [10]. Furthermore, among 2351 14-year-old children in North West England, 53 % had at least one tooth surface with exposed dentin [11]. These surveys reflect that tooth wear in adolescents is common.

In China, data about the prevalence of tooth wear are scarce. The purposes of this study were to describe the tooth wear status of 12- and 15-year-old adolescents from Central China and to investigate the relative effects of risk indicators on tooth wear.

## Methods

The study protocol was approved by the Ethics Committee, School & Hospital of Stomatology of Wuhan University (Wuhan, China).

### Sampling

**Fig. 1:**
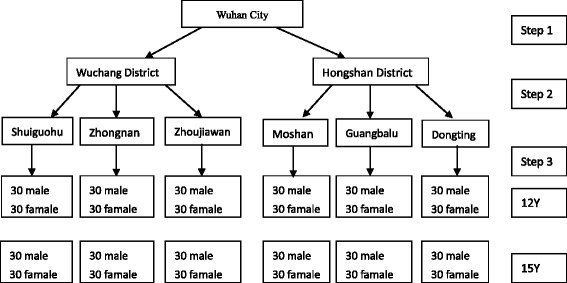
Schematic illustration for multistage sampling

The cross-sectional survey was completed in August 2014. The samples were 12- and 15-year-old adolescents living in Wuhan City for more than 6 months. We used a multi-stage stratified sampling method in Wuhan City (Fig. 1) and estimated the sample sizes based on expected prevalence. The sizes of the samples were calculated to have a 5 % acceptable margin of error and an alpha level of 0.05; assuming that the prevalence of tooth wear in the 12- and 15-year-old groups was 30 % [12] with a 95 % confidence interval (CI), the sample size required was 322. Finally, each group actually consisted of 360 participants. The sample for this study consisted of urban residents from 6 survey spots. First, two districts (Wuchang District and Hongshan District) were chosen by random selection from a total of 13 districts. Then, three resident communities were randomly chosen from 191 community resident committees in Wuchang and 124 community resident committees in Hongshan respectively. In the third stage, 60 subjects were randomly selected from each community among each of the two age groups, half male and half female. Finally, we had 720 participants in total. The subjects were included based on the following criteria, which required them to be 12 and 15 years of age, able to understand this study and to understand and read a questionnaire and also to be willing to cooperate, to sign a relevant informed consent letter, and to maintain good compliance. Participants were excluded when they could not understand this study, had oral disease requiring medication, or had systemic diseases that could impact the integrity of the data and/or the safety of the participant.

Each participant signed an informed consent letter before examination.

### Interview

A self-administered questionnaire was conducted in Chinese, consisting of questions about the frequency of acidic food intake (fresh fruit, fruit juices, vegetable juices, carbonated drinks, yogurt, coffee, wine, pickled vegetables, vinegar), medicines (vitamin C, aspirin, amphetamine, diazepam), general health (symptoms of reflux, vomiting, eating disorders), digestive system disease (gastro-esophageal reflux disease, gastritis, xerostomia), frequency of swimming in the summer, chewing habits, dietary factors, oral health-associated preventive behaviors and the family’s social-economic class.

### Clinical examination

The two examiners in this study were trained and calibrated using a group of samples, under the guidance of Bartlett [13] prior to the formal examination. The inter-examiner kappa scores of the two examiners were both greater than 0.70 at the end of training. To test the intra-examiner reliability during the survey period, 5 % of the subjects (6 individuals) were randomly chosen on each day to be re-examined. The second examination occurred after two subjects or at least after 15 min. The intra-examiner kappa values of the two examiners were 0.80 and 0.82.

After completing the questionnaire, the two examiners performed all of the assessments after drying the tooth surfaces with cotton rolls under artificial light without amplification or constringent air. The Basic Erosive Wear Examination (BEWE) has only four primary classifications: no surface loss (0), initial loss of enamel surface texture (1), distinct defect and hard tissue loss (dentin) over less than 50 % of the surface area (2) and hard tissue loss over more than 50 % of the surface area (3) that cannot be managed without a complicated score criteria. Therefore, this study used a modified version of the BEWE. The modified BEWE (Table 1) includes an score for wearing orthodontic appliances, caries or restoration on more than 25 % of the surface area, partial eruption, trauma, and crowns that cannot be assessed, as well as another score for missing teeth. In addition, two scores were used to assess the prevalence of dentin exposure. The buccal, cervical, occlusal/incisal and lingual surfaces of all of the teeth, except for the third molar, were examined for lesions.

**Table 1:** The modified Basic Erosive Wear Examination (BEWE) index used in this study

Score	Enamel wear	Dentine exposure
0	No tooth wear	No
1	Initial loss of surface texture	Yes
2	Distinct defect, hard tissue loss <50 % of the surface area	
3	Hard tissue loss ≥50 % of the surface area	
8	Orthodontic appliances, caries or restoration ≥25 % of the	
	Surface area, partial eruption, trauma, crown, can’t assessed	
9	Missing	

(We added the enamel wear scores 8 and 9, and dentine exposure scores 0 and 1.)

### Statistical analysis

Statistical Package for the Social Sciences software (SPSS; Chicago, IL, USA), version 21, was used to perform the statistical analyses.

Each subject was characterized by the highest BEWE score (except 8 and 9). The prevalence of tooth wear was the proportion of individuals with teeth of BEWE ≥2 in the study population. The prevalence of dentin exposure was the proportion of individuals with teeth with a dentin score of 1. All of the indicators involved in the questionnaire were explanatory variables, and BEWE index of 2–3 was the dependent variable in the Chi-square test and binary logistic regression analysis. To assess the associations between the consumption of acidic food and fruit juices and tooth wear, the sum score was calculated (score 9–54). In the final analysis, some of the various indicators were categorized empirically.

All of the teeth in six sextants were checked in this study, and the sum of the highest score (except 8 and 9) for each sextant (17–14, 13–23, 24–27, 37–34, 33–43, 44–47) was calculated as an indication for clinical management. The association between each variable and tooth wear was tested using the Chi-square test, and the independent samples *t*-test was used for comparison of the mean index of the two age groups. The level of statistical significance was set at 0.05. All of the explanatory variables found to be significant by Chi-square analysis were included in binary logistic regression analysis to determine the independent effect of each explanatory variable on the dependent variable while controlling for other variables. Odds ratios (ORs) with 95 % confidence intervals (95 % CIs) were calculated for the discrete variables in the logistic regression model. The level of statistical significance was set at 0.05.

## Results

The prevalence of tooth wear and dentin exposure was shown in Table 2. The prevalence rates of tooth wear were 18.6 and 89.4 %, with mean indices of 1.19 (SD = 0.41) and 1.97 (SD = 0.44), in 12- and 15-year-old adolescents, respectively. The prevalence of dentin exposure was 1.9 and 5.6 %, respectively. The 15-year-old group had significantly higher percentages of tooth wear and dentin exposure than the 12-year-old group (*P* < 0.001 and *P* = 0.011), and the mean index of the 15-year-old group was significantly higher than of the 12-year-old group (*p* < 0.001). Neither tooth wear nor dentin exposure was associated with gender.

**Table 2:** The prevalence and mean score of tooth wear according to gender

	*N*	Prevalence of TW	Prevalence of DE	Mean score of BEWE (±SD)
		*N* (%)	*N* (%)	
12 years				
Boy	180	35 (19.4)	7 (3.9)	
Girl	180	32 (17.8)	0 (0)	
Total	360	67 (18.6)	7 (1.9)	1.19 (±0.41)
15 years				
Boy	180	166 (92.2)	10 (5.6)	
Girl	180	156 (86.7)	10 (5.6)	
Total	360	322 (89.4)	20 (5.6)	1.97 (±0.44)

*TW* Tooth Wear, *DE* Dentine Exposure, *BEWE* Basic Erosive Wear Examination

In the present study, the sum of six sextants’ highest BEWE score of the two age groups had a significant difference (*p* < 0.001). As observed from Table 3, the most common rank was 3–8 in both the 12- and 15-year-old groups, indicating that the majority of the adolescents were at a low risk level.

**Table 3:** The number of adolescents at different rank of Basic Erosive Wear Examination (BEWE) score sum

	Score sum			
	0–2	3–8	9–13	14-
12 years				
Boy	0	180	4	0
Girl	1	175	0	0
Total	1	355	4	0
15 years				
Boy	0	127	55	0
Girl	1	133	42	2
Total	1	260	97	2

Tables 4, 5, 6, 7 show the associations between the percentages of tooth wear and various factors, assessed by the Chi-square test. The occurrence of tooth wear was significantly associated with age, holding soft drinks and fruit juices in the mouth, consuming drinks immediately after sports, swimming in the summer, taking aspirin and diazepam, having reflux, eating hard food, and unilateral chewing, as well as frequency and duration of brushing and social-economic class.

**Table 4:** The percentages of respondents with tooth wear (BEWE2-3) according to demographics and oral hygiene factors

	*N*	Tooth wear		*P*
		BEWE2-3	%	
Age				<0.001
12 years	360	67	18.6	
15 years	360	322	89.4	
Gender				0.331
Boy	360	201	55.8	
Girl	360	188	52.2	
Social-economic class				0.045
Low (scores 1–5)	283	166	58.7	
High (scores 6–10)	437	223	51.0	
Frequency of brushing				0.004
Twice or more daily	543	277	51.0	
Once or less daily	177	112	63.3	
Duration of brushing				0.038
> 2 min	218	105	48.2	
≤ 2 min	502	284	56.6	
Toothbrush bristle				0.064
Soft bristles	381	196	51.4	
Medium	223	130	58.3	
Hard bristles	35	24	68.6	
Toothbrushing method				0.714
Mixed brushing	459	245	53.4	
Horizontal brushing	103	54	52.4	
Vertical brushing	135	77	57.0	
Fluoride toothpaste				0.077
Yes	231	117	50.6	
No	63	24	38.1	
Accumulated use time				0.987
> 1 year	142	72	50.7	
< 1 year	89	45	50.6	
Toothbrush				0.778
Manual toothbrush	621	336	54.1	
Electric toothbrush	27	16	59.3	
Both	72	37	51.4	
Frequency of changing toothbrush				0.967
> 3 months	354	191	54.0	
< 3 months	366	198	54.1

**Table 5:** The relationship between tooth wear (BEWE2-3) and dietary factors

	*N*	Tooth wear		*P*
		BEWE2-3	%	
Fruit juices/soft drinks/acid food				0.142
Score 9–22	352	200	56.8	
Score 23–54	368	189	51.4	
Taking drinks before sleep				0.219
Never	370	188	50.8	
Rarely	240	133	55.4	
Sometimes	74	46	62.2	
Often	36	22	61.1	
Holding drinks in mouth				<0.001
Never	433	205	47.3	
Rarely	210	131	62.4	
Sometimes	58	40	69.0	
Often	19	13	68.4	
Drinking with straw				0.464
Never	138	68	49.3	
Rarely	255	135	52.9	
Sometimes	219	126	57.5	
Often	108	60	55.6	
Drinking immediately after sport				<0.001
Never	160	72	45.0	
Rarely	247	122	49.4	
Sometimes	197	115	58.4	
Often	116	80	69.0	
Dry mouth				0.578
Never	159	82	51.6	
Rarely	241	126	52.3	
Sometimes	168	92	54.8	
Often	152	89	58.6	
Frequency of tea consumption				0.681
< 2–6 times weekly	404	221	54.7	
> 2–6 times weekly	316	168	53.2	
Eating hard food				0.032
Never	153	90	58.8	
Rarely	301	144	47.8	
Sometimes	186	105	56.5	
Often	80	50	62.5

**Table 6:** The relationship between tooth wear (BEWE2-3) and general health

	*N*	Tooth wear		*P*
		BEWE2-3	%	
Vitamin C supplements				0.790
Never	273	148	54.2	
Rarely	233	121	51.9	
Sometimes	137	75	54.7	
Often	77	45	58.4	
Taking Aspirin				0.002
Never	517	260	50.3	
Rarely	160	97	60.6	
Sometimes	31	21	67.7	
Often	12	11	91.7	
Taking Amphetamine				0.111
Never	656	348	53.0	
Rarely	47	27	57.4	
Sometimes	10	8	80.0	
Often	7	6	85.7	
Taking Diazepam				0.043
Never	694	369	53.2	
Rarely	18	12	66.7	
Sometimes	4	4	100	
Often	4	4	100	
Reflux				0.012
Never	490	245	50.0	
Rarely	166	107	64.5	
Sometimes	51	29	56.9	
Often	13	8	61.5	
Vomiting				0.601
Never	484	267	55.2	
Rarely	196	104	53.1	
Sometimes	32	15	46.9	
Often	8	3	37.5	
Eating disorder				0.269
Never	478	265	55.4	
Rarely	177	96	54.2	
Sometimes	49	20	40.8	
Often	16	8	50.0	
Gastro esophageal reflux disease				0.850
No	381	177	46.5	
Yes	7	3	42.9	
Gastricism				0.161
No	368	171	46.5	
Yes	32	19	59.4	
Xerostomia				0.725
No	376	176	46.8	
Yes	12	5	41.7

**Table 7:** The relationship between tooth wear (BEWE2-3) and life style factors

	*N*	Tooth wear		*P*
		BEWE2-3	%	
Frequency of swimming in summer				<0.001
Never/rarely	222	144	64.9	
1–2 times weekly	282	152	53.9	
3–4 times weekly	156	70	44.9	
> 5 times weekly	60	23	38.3	
Clenching teeth automatically				0.217
Never	474	250	52.7	
Rarely	163	87	53.4	
Sometimes	60	35	58.3	
Often	23	17	73.9	
Sleep bruxiam				0.332
Never	589	315	53.5	
Rarely	82	47	57.3	
Sometimes	33	21	63.6	
Often	16	6	37.5	
Chewing habits				<0.001
Both	469	230	49.0	
Left	85	53	62.4	
Right	119	81	68.1	

Table 8 shows the proportion of participants with BEWE score of 2 or 3 relative to oral hygiene and dietary factors using binary logistic regression analysis. Only statistically significant associations are presented. The results of logistic regression analysis demonstrated a higher prevalence in participants who brushed their teeth once daily or less (OR = 1.5, *p* = 0.043) and with a duration of brushing of less than 2 min (OR = 1.5, *P* = 0.030); in addition, subjects drinking soft drinks and fruit juices immediately after sports, taking aspirin, having reflux and chewing unilaterally also tended to have a high likelihood of experiencing tooth wear. Nevertheless, swimming in the summer was found to be negatively correlated with the occurrence of tooth wear in 12- and 15-year-old adolescents from Central China.

**Table 8:** Logistic regression analyses of odds for tooth wear among Central China adolescents

	*P*	OR	95 % CL	
			lower	upper
Drinking immediately after sport				
Never	0.007	1	1	
Rarely	0.307	1.2	0.811	1.948
Sometimes	0.025	1.7	1.070	2.716
Often	0.001	2.5	1.415	4.320
Frequency of swimming in summer				
Never/rarely	0.002	1	1	
1–2 times weekly	0.034	0.7	0.441	0.968
3–4 times weekly	0.000	0.4	0.280	0.699
> 5 times weekly	0.008	0.4	0.205	0.784
Taking Aspirin				
Never	0.026	1	1	
Rarely	0.128	1.4	0.915	2.035
Sometimes	0.067	2.2	0.947	5.074
Often	0.033	11.1	1.209	102.425
Reflux				
Never	0.033	1	1	
Rarely	0.003	1.8	1.223	2.734
Sometimes	0.863	1.1	0.558	2.005
Often	0.966	1.0	0.298	3.540
Chewing habits				
Both	0.003	1	1	
Left	0.054	1.6	0.992	2.708
Right	0.002	2.2	1.289	3.191
Frequency of brushing	0.043			
Twice or more daily		1	1	
Once or less daily		1.5	1.012	2.227
Duration of brushing	0.030			
> 2 min		1	1	
≤ 2 min		1.5	1.039	2.099

## Discussion

The complex nature of tooth wear leads to difficulties in the management of wear studies. There are many tooth wear assessment criteria, most of which are based on quantitative and qualitative analyses. There are some Tooth Wear Indexes (TWIs) that have been used more frequently [6, 8, 10, 14], such as the Smith and Knight tooth wear index [15]. However, this index lacks any standardization, and it is difficult to reconcile both clinical and experimental imperatives [13]. Furthermore, the different classifications of the assessment criteria have failed to enable direct comparisons between various studies [16]. Hence, the Basic Erosive Wear Examination (BEWE) was designed by Bartlett et al. to provide a simple scoring system so that it could be used with the diagnostic criteria of all of the existing indices to convert their results into one unit, namely the BEWE score sum. In this study, the most common rank of BEWE score sum was 3–8. As Bartlett suggested, the corresponding management is oral hygiene and dietary assessment, advice, routine maintenance and observation, and repetition at 2-year intervals [13]. The literature has revealed that the BEWE was a convenient index to use, with sufficient sensitivity and specificity [17].

There are few systematic data on tooth wear from China. This study aimed to describe the situation of tooth wear in China and to assess the relative risk indicators using the BEWE index. We checked 720 12- and 15-year-old adolescents in Hubei Province, which is located in the heart of Central China. In this cross-sectional study, the severity and related indicators of tooth wear were assessed. The prevalence of tooth wear was 18.6 % in 12-year-old adolescents, which was lower than that in 12-year-old adolescents in Brazil [10, 18], Australia [19] and Libya [20] but higher than in US [21] and the study by Peres in Brazil [22]. It is important to note that the prevalence was much lower than that in the study by Hou et al. in Beijing, China [23], and that in the study by Wang et al. in Southern China [24]. The prevalence among 15-year-old adolescents was 89.4 %, which was higher than the study by Sanhouri et al. in Khartoum State, Sudan (74 %) [25]. The large variation might be due to the differences in evaluation criteria and statistical measurement criteria.

There is substantial evidence revealing that the consumption of beverages, particularly carbonated soft drinks, is a risk factor for dentin erosion in adolescents [26–28]. An in vitro study suggested that beverage consumption had the potential to erode both enamel and root surfaces [29]. In this study, no significant relationship was found between tooth wear and the consumption of soft drinks and fruit juice. It is not clear whether this result was because the consumption of soft drinks and fruit juice is uncommon among 12- and 15-year-old adolescents in Wuhan Hubei. However, the odds ratio of those who often drank beverages immediately after sports was 2.5 compared with those who never did. In the literature, the influence of saliva on acidic drinks has been determined. An acidic drink was quickly buffered in the mouth of normal subjects within minutes and returned to salivary levels rapidly when the beverage was finished [30]. Subjects after participating in sports had low saliva flow because of the body’s dehydration [31], indicating that they would take a longer period of time to consume carbonated beverages, and their teeth would undergo longer exposure to low pH value, placing them at a high level of risk for developing erosion.

Previous study has confirmed that participants who brushed their teeth twice or more per day had a high frequency of tooth wear [32]. In this study, 177 subjects brushed their teeth less than twice daily, and their risk was 1.5 times higher than that of people who brushed their teeth twice daily or more. At the same time, the odds ratio for a duration of brushing of less than 2 min was 1.5 compared with that for a duration of more than to minutes. The difference might have been caused by the different age group because the former study’s participants were much older (standard age groups of 35–44 and 65–74 years old). The results of this study might have occurred because the plaque on tooth surfaces for long periods of time can utilize carbon to produce acid that erodes the enamel surfaces [33] and effective tooth brushing can reduce the attachment and accumulation of bacterial plaque [34], while tooth brushing less than twice daily or less than two minutes at a time might increase the risk in adolescents.

There were a few subjects who often took aspirin and their risk was 11.1 times greater than people who never took aspirin. This finding might have been caused by acetylsalicylic acid which is a low PH medicine. An in vitro study [35] exposed extracted teeth in 5 ml of water with 500-mg aspirin tablet, and the results demonstrated that even a short period of exposure could lead to measurable and observable erosion of the tooth structure on all of the exposed surfaces. Furthermore, longer exposure caused more erosion. McCracken et al. [36] and Grace et al. [37] reported clinical cases of tooth erosion caused by aspirin, providing important support for our result. In the present study, reflux was a risk factor for tooth wear, which is in agreement with the results of previous investigations by Smith et al. [6] and Gregory-Head et al. [38].

Previous studies have shown that masticatory preference was more apparent when chewing hard food [39] because hard food requires more effort to chew than softer food and can cause more laterality [40]. Patients with unilateral chewing will choose the side with greater force to masticate hard food, and masticatory efficiency is equal when using both sides synchronically [41], causing more tooth wear to the preferred chewing side. The present study revealed that unilateral chewing was positively significant for the prevalence of tooth wear. The odds of unilateral chewing with the left and right sides were 1.6 and 2.2, respectively, in subjects compared with subjects chewing with both sides. In addition, researches have indicated that swimming in improperly chlorinated pool could raise the risk of tooth wear because the water with acidic pH could cause erosion of dental enamel [42, 43]. However, there was different result in our study, which showed that swimming in swimming pools in the summer had negatively significant effects on tooth wear. Perhaps it was only because the subjects of previous studies were competitive swimmers who underwent intensive training for long periods of time, while the adolescents in this study swam only for entertainment and for shorter periods of time.

This study had limitations because of the cross-sectional study design. The longitudinal study should be done in the future for better elucidation of the risk factors of tooth wear.

## Conclusions

This study showed that tooth wear in 12- and 15-year-old adolescents was a significant problem. At the same time, it confirmed that the etiology of tooth wear was multi-factorial. Drinking soft drinks and fruit juices immediately after sports, taking aspirin, having reflux, unilateral chewing, brushing the teeth once daily or less and having a duration of brushing less than 2 min increased the risk of tooth wear, and swimming in the summer for entertainment reduced the association with tooth wear.
